# Daily activities and health-related quality of life in patients with chronic obstructive pulmonary disease: psychological determinants: a cross-sectional study

**DOI:** 10.1186/1477-7525-11-190

**Published:** 2013-11-05

**Authors:** Saskia WM Weldam, Jan-Willem J Lammers, Rogier L Decates, Marieke J Schuurmans

**Affiliations:** 1 Department of Respiratory Diseases, Division Heart & Lungs, University Medical Center Utrecht, HP E03.511, PO Box 85500, Utrecht 3508 GA, the Netherlands; 2 Department of Rehabilitation, Nursing Science & Sports, University Medical Center Utrecht, Utrecht, the Netherlands

**Keywords:** Daily Activities (DA), COPD, Chronic obstructive pulmonary disease, Depressive symptoms, Functional performance, Illness perceptions, Proactive coping, Health-related quality of life

## Abstract

**Background:**

Chronic obstructive pulmonary disease (COPD) patients are confronted with reduced daily activities (DA) and reduced health-related quality of life (HRQoL) caused by dyspnea and systemic effects such as skeletal muscle dysfunction and co-morbidities. To understand the complexity of living with COPD, it is important to understand which factors, in addition to physical functioning, are associated with DA and HRQoL. In this study, we explored the extent to which the combination of illness perceptions, proactive coping, and depressive symptoms contribute to DA and HRQoL in COPD patients.

**Method:**

In a cross-sectional study in primary care, 90 COPD patients (GOLD I-III) completed questionnaires: the Brief Illness Perception Questionnaire, the Utrecht Proactive Coping Competence scale, the Centers for Epidemiologic Studies Depression scale, the Medical Research Council dyspnea scale, the Functional Performance Inventory (FPI), and the Clinical COPD Questionnaire (CCQ). The analyses were performed with multiple linear regression analyses.

**Results:**

More adequate and positive illness perceptions (β = .61, p < .001) and less depressive symptoms (β = .21, p = .010) were associated with better HRQoL (CCQ). Significant relations between psychological factors and DA were not found.

**Conclusion:**

The results of this study demonstrate that psychological factors are related to HRQoL, but not to DA. These results contribute to understanding the complexity of living with COPD and provide starting points for the development of interventions focusing on psychological factors to support COPD patients in disease management.

## Background

Chronic obstructive pulmonary disease (COPD) is one of the leading causes of morbidity and mortality in the world [1]. The prevalence of the social and economic burden that results from this illness continues to increase [1,2]. Previous research on COPD has shown that COPD patients are confronted with daily life limitations, reduced daily activities (DA), and reduced health-related quality of life (HRQoL) caused by complaints such as dyspnea, skeletal muscle dysfunction, and co-morbidities [2-7].

In the last decade, in the Netherlands and in many other countries, care for patients with COPD has increasingly moved from hospitals to primary care [8]. According to the COPD guidelines in the Netherlands, general practitioners and practice nurses play a key role in care for COPD patients in primary care [9]. Education and counseling are the most important tasks of practice nurses. These nurses have become essential in the integrated care for COPD patients in the Netherlands [8]. Although research is being conducted among COPD populations in primary care [10-12], much of the research on COPD patients is conducted in hospital-based populations.

To understand the complexity of living with COPD, it is important to understand which factors, in addition to physical functioning and complaints, are associated with DA and HRQoL [13,14]. Psychological factors, such as illness perceptions, depressive symptoms, and ability to cope with the illness, may complicate living with COPD and participation in everyday activities [15-18].

According to the Common Sense Model by Leventhal [19], patients’ beliefs and perceptions about illness guide individuals’ efforts to cope with illness. Studies of the rehabilitation of COPD patients [20] and patients who attended outpatient clinics [21] show that COPD patients with positive beliefs about the impact of their disease on daily life and those with more positive thoughts about the effect of their treatment have a better HRQoL than patients who have more negative perceptions. Concerns about COPD are negatively related to walking test results [22] and general functioning [23]. Although research is being conducted among COPD populations in primary care [11,12,24], knowledge of the associations among illness perceptions, HRQoL, and DA in COPD patients in primary care is scarce.

To adjust to life with COPD and to prevent further physical deterioration, it is important that patients participate in everyday activities and physical exercises and that they anticipate potential threats [25]. In this respect, proactive coping, which refers to efforts undertaken to prevent or modify a potential threat to a person’s health, may play an important role [26,27]. Some studies show that people who have the ability to anticipate and address potential threats to their health have better outcomes in goal achievement and general health behavior [27,28]. However, proactive coping has not been studied in COPD patients.

Furthermore, multiple studies covering a variety of medical settings have shown that depressive symptoms are prevalent among COPD patients and that these symptoms are related to inactivity and lower HRQoL [29-31]. Prevalence estimates of depressive symptoms vary (10%-42%) due to the use of varied measurement tools [30,31]. A meta-analysis revealed that the prevalence of depressive symptoms was two times higher in COPD patients than in healthy controls [31].

In this cross-sectional study, we will explore the extent to which the *combination* of these psychological factors (illness perceptions, proactive coping, and depressive symptoms) contribute to DA and HRQoL in COPD patients in primary care. We hypothesize that positive illness perceptions (i.e., positive beliefs about the effects of the illness on daily life), adaptive proactive coping competencies, and low levels of depressive symptoms are associated with better DA and better HRQoL in COPD patients.

## Method

### Study design and participants

This cross-sectional study was conducted in primary care settings in the Netherlands. COPD patients from ten general practices throughout the Netherlands who regularly visited the consulting hours of the practice nurses were asked to participate. To be eligible for inclusion, patients had to be diagnosed with COPD GOLD grades I, II, or III [25] and be physically and mentally able to complete the questionnaires. The patients were excluded if they participated in another study or if they had COPD GOLD grade IV or a primary diagnosis of asthma. The study was approved by the Medical Ethical Committee of the University Hospital Utrecht (UMC Utrecht), and all participants provided written informed consent.

### Procedure

Practice nurses working in the participating general practices included the COPD patients who visited their consulting hours. After providing written informed consent, the participating patients completed the questionnaires at home and returned them to our center. Data were collected from June 2010 to April 2011.

### Measures

#### Illness perceptions

The Brief Illness Perception Questionnaire (B-IPQ) [32] was used to assess illness perceptions concerning consequences, timeline, personal control, treatment control, identity, concern, understanding, emotional representations, and causal representations. The B-IPQ is a self-administered scale consisting of nine items (range: 0–10). To assess the degree to which COPD is perceived as threatening or benign, an overall summary score was computed. Three items (personal control, treatment control, and understanding) were reversed and added to the other items. A higher score reflects a more threatening view of COPD. A lower score reflects a more positive and adaptive view of COPD.

### Proactive coping

The Utrecht Proactive Coping Competence questionnaire (UPCC) [33] was used to assess individuals’ competency with regard to the various skills associated with proactive coping. The questionnaire is self-administered and consists of 21 items that together form one factor. The response options range from 1 (“not competent”) to 4 (“very competent”). A higher score reflects more proactive coping competencies.

### Depressive symptoms

The Centers for Epidemiologic Studies Depression Scale (CES-D scale) [34] was used to assess depressive symptoms. The CES-D scale was not developed to diagnose depression; rather, it aims to identify depressive symptoms. It is a self-administered questionnaire consisting of 20 items related to situations during the previous week. The response options range from 0 (“seldom”) to 3 (“mostly/always”). High scores indicate more depressive symptoms.

### Dyspnea

The Medical Research Council (MRC) scale [35] was used to assess dyspnea. Dyspnea was rated by the patient with five increasing scores with option ranges from 0 (“not breathless except for exertion”) to 4 (“too breathless to leave house or breathless when dressing or undressing”).

### Daily activities

Daily activities (DA) were measured by the Functional Performance Inventory (FPI) [36]. This self-administered questionnaire measures the extent to which people engage in their usual day-to-day activities. The FPI consists of 65 items and has six subscales: body care (9 items), household maintenance (21 items), physical exercise (7 items), recreation (11 items), spiritual activities (5 items), and social activities (12 items). Response options range from 1 (“the activity can be performed easily, with no difficulty at all”) to 4 (“the activity is no longer performed for health reasons”) with an option to answer “the activity is not performed for reasons other than health”. In the total score, the items are recoded to indicate that high scores represent high performance.

### Quality of life

The Clinical COPD Questionnaire (CCQ) was used to measure HRQoL [37]. The CCQ is a self-administered questionnaire and consists of ten questions covering three domains: functional state, symptoms, and mental state. Response options range from 0 (“no limitations/asymptomatic”) to 6 (“totally limited/extremely symptomatic”).

Data were collected on height and weight (according to the local general practitioner registry), and BMI was calculated as weight (kg)/height (m) squared. Data on pulmonary function (FEV1 in liters, FEV1% predicted, FVC in liters, and FEV1/FVC ratio), co-morbidities (Charlson co-morbidity Index) [38], and demographic variables (age, gender, illness duration, medication, education level, working status, smoking status, and marital status) were also collected.

### Analyses

Descriptive statistics (frequencies, mean, and standard deviation) were used to present patients’ background and medical characteristics. Linear regression analyses (adjusted for the confounders age, gender, dyspnea, FEV_1_, smoking status, and co-morbidity) were applied to quantify the associations between illness perceptions, proactive coping, and depressive symptoms, on the one hand, and DA and QoL, on the other hand. First, a crude model was developed with the separate psychological determinants (illness perceptions, proactive coping, and depressive symptoms) (model 1). In the second model (model 2), the three psychological variables were combined in one model and corrected for the confounders age and gender. The third model (model 3) was additionally adjusted for dyspnea, FEV_1_, smoking status, and co-morbidity. In the regression models, the standardized βs were used to compare the strength of the various independent variables. The explained variance per model was analyzed. Because the amount of missing data was only 3%, a complete case analysis was performed. All analyses were performed with Statistical Package for the Social Sciences (SPSS 20.0 for Windows).

## Results

### Study sample

Sample characteristics are presented in Table 1. A total of 98 patients completed the questionnaires. In the analyses, eight patients were excluded because they appeared to have GOLD stage 0 with an FEV_1_/FVC ratio >70%. Most patients had GOLD grade II (n = 60, 67%). Of the patients in this study, 46% were women, and mean age of the sample was 65 (SD 9.0). Most of the patients had a medium educational level (n = 56; 62%), and approximately half of the patients were retired (51%). Of the non-retired population (n = 44), 45% (n = 20) had a full-time job.

**Table 1:** Patient characteristics

**Variables**		**Total population (N = 90)**
*Gender*		
	Male	49 (54.4%)
	Female	41 (45.6%)
*Age, years*		
	Mean	65.19 (SD 9.0)
	≤ 50	5 (5.6%)
	51–60	26 (28.9%)
	61–70	31 (34.4%)
	71–80	23 (25.6%)
	> 80	5 (5.6%)
*Years diagnosed with COPD*		8.13 (SD8.11)
*Disease severity*		
	GOLD^a^ I	18 (20.0%)
	GOLD^a^ II	60 (66.7%)
	GOLD^a^ III	12 (13.3%)
	FEV_1_ mean	1.86 (1.02-3.70) (SD .59)
	FEV_1_% pred	67.0 (36.54-101.22) (SD 14.4)
*Education level*^*b*^		
	Low	13 (14.4%)
	Medium	56 (62.3%)
	High	21 (23.3%)
*Retired*		46 (51.1%)
*Paid work*		20 (2.2%)
*Marital status*		
	Married	59 (65.6%)
	Widowed	8 (8.9%)
	Divorced	8 (8.9%)
	Single	15 (16.7%)
*Smoking status*		
	Current smoker	36 (40.0%)
	Former smoker	49 (54.4%)
	Never smoked	5 (5.6%)
*Medication use*		82 (91.1%)
*Polypharmacy*^*c*^		28 (31.1%)
*Charlson comorbidity index >1*		28 (31.1%)

^a^Global Initiative for Chronic Obstructive Lung Disease.

^b^Categories are based on the International Standard Classification of Education (ISCED).

^c^Defined by using five or more different types of medication.

The means, standard deviations, range of illness perceptions, proactive coping, depressive symptoms, dyspnea, FEV_1_, DA, and HRQoL are presented in Table 2.

**Table 2:** **Means, standard deviations, range of illness perceptions (B-IPQ), proactive coping (UPCC), depressive symptoms (CES-D), dyspnea (MRC-dyspnea), FEV**_**1**_**, daily activities (FPI), and health-related quality of life (CCQ) (N = 88–90)**

	**Mean (SD)**	***Range***	***Ref Range***
1. B-IPQ	33.21(11.3)	10 – 68	0 – 100
2. UPCC	3.0 (0.4)	1.9 – 4.0	1.0 – 4.0
3. CES-D	10.2 (7.4)	0 – 42	0 – 60
4. MRC–dyspnea	1.7 (1.0)	0 – 5	0 – 6
5. FEV_1_ (liters)	1.86 (0.59)	1.0 – 3.7	
6. FEV% pred	67.0 (14.4)	36.54 – 101.22	
7. FPI	1.80 (0.4)	1.0 – 2.9	1 – 3
8. CCQ	1.4 (0.8)	0.0 – 3.8	0 – 6

B–IPQ = Brief Illness Perception Questionnaire, UPCC = Utrecht Proactive Coping Competence scale, CES–D = Centers for Epidemiologic Studies Depression scale, MRC dyspnea = Medical Research Council dyspnea scale,

FEV_1_ = Forced Expiratory Volume in one second, FEV% pred = Forced Expiratory Volume Percentage from predicted, FPI = functional Performance Inventory, CCQ– = Clinical COPD Questionnaire.

The average on the FPI scale was 1.8 (range: 1.0–2.9, reference range 1–3), indicating intermediate levels of DA [36]. The average on the CCQ was 1.4 (range: 0–3.8, reference range 0–6), meaning that the HRQoL was good, on average [37].

The mean of illness perceptions was 33.2 (range: 10–68, reference range 0–100), indicating that the patients had, on average, a more positive and adequate perception of their COPD [32]. The mean of proactive coping was 3.0 (range: 1.8–4.0, reference range 1.0–4.0), which is similar to healthy adults [33]. The mean of depressive symptoms was 10.2 (range: 0–42, reference range 0–60). Twenty patients had a score >16, indicating that 22.2% of the patients studied were possible cases of depression (three patients with GOLD grade I, 15 with GOLD grade II, and two patients with GOLD grade III) [34].

### Regression analyses

#### Daily activities

As shown in Table 3, in the univariate models (model 1), illness perceptions (β = -.25) and depressive symptoms (β = -.21) were associated with DA measured by the Functional Performance Inventory. Proactive coping was not associated with DA. In model 2, where the psychological determinants illness perceptions, proactive coping, and depressive symptoms were combined and corrected for the confounders age and gender, only illness perceptions (β = -.22) and age (β = -.25) were associated with DA. In the third model, the combination of the psychological determinants illness perceptions, proactive coping, and depressive symptoms were corrected for the confounders age, gender, dyspnea, FEV_1_, smoking status, and co-morbidity. The psychological determinants were not associated with DA. Only age (β = -.21) and dyspnea (β = -.29) were significantly associated with DA (Table 3).

**Table 3:** Regression model of illness perceptions (B-IPQ), proactive coping (UPCC), and depressive symptoms (CES-D) and the dependent variable daily activities (FPI) (N = 87)

	**Model 1 (Block 1) univariate**			**Model 2 (Block 1 and 2)**			**Model 3 (Block 1, 2, 3)**		
	**R2**	**β**	**95% CI**	**R2**	**β**	**95% CI**	**R2**	**β**	**95% CI**
*Block 1*				.15			.24		
B-IPQ	.06	-.25^*^	-.02 - .00		-.22*	-.02 - .00		-.11	-.01 - .00
UPCC	.03	.182	-.03 - .36		.04	-.17 - .24		.00	-.20 - .21
CES-D	.05	-.21*	-.02 - .00		-.11	-.02 - .01		-.09	-.02 - .01
*Block 2*									
Age					-.25*	-.02 - .00		-.21*	-.02 - .00
Gender					-.03	-.19 - .14		.00	-.16 - .17
*Block 3*									
MRC dyspnea								.29**	-.20 - -.03
Fev_1_								.12	-.00 - .01
Smoking status								.02	-.15 - .18
Co-morbidities								-.06	-.22 - .13

* P ≤ 0.05 ** P ≤ 0.01.

Model 1: Crude model (univariate) separate: Illness perceptions (B-IPQ), Proactive coping (UPCC), Depressive symptoms (CES-D).

Model 2: B-IPQ, UPCC and CES-D corrected for confounders age and gender.

Model 3: B-IPQ, UPCC and CES-D additionally adjusted for dyspnea, FEV_1_, smoking status, and co-morbidities.

### Quality of life

As shown in Table 4, illness perceptions (β = .61) and depressive symptoms (β = .21) were associated with HRQoL (CCQ) in the univariate models (model 1), in model 2 (the combination of illness perceptions, proactive coping, and depressive symptoms, corrected for the confounders age and gender), and in model 3, which is corrected for age, gender, dyspnea, FEV_1_, smoking status, and co-morbidity. Thus, COPD patients with a more positive view of their illness and fewer depressive symptoms had a better HRQoL. Illness perceptions and depressive symptoms together with dyspnea explained 60% of the variance in HRQoL. Proactive coping was not associated with HRQoL.

**Table 4:** Regression model of illness perceptions (B-IPQ), proactive coping (UPCC), and depressive symptoms (CES-D) and the dependent variable health-related quality of life (CCQ) (N = 87)

	**Model 1 (Block 1) univariate**			**Model 2 (Block 1 and 2)**			**Model 3 (Block 1, 2, 3)**		
	**R2**	**β**	**95% CI**	**R2**	**β**	**95% CI**	**R2**	**β**	**95% CI**
*Block 1*				.55			.60		
B-IPQ	.48	.69***	.04 - .06		.68***	.039 - .06		.61***	.03 - .06
UPCC	.04	-.20	-.80 - .03		.09	-.142 - .513		.12	-.08 - .55
CES-D	.10	.32**	.014 - .06		.22**	.008 - .049		.21**	.01 - .05
*Block 2*									
Age					.01	-.013 - .015		-.00	.02 - .01
Gender					.03	-.205 - .311		-.01	-.26 - .25
*Block 3*									
MRC dyspnea								.26***	.09 - .35
Fev_1_								-.03	-.01 - .01
Smoking status								-.00	.27 - .25
Co-morbidities								-.04	-.35 - .19

** P ≤ 0.01 ***P ≤ 0.001.

Model 1: Crude model (univariate) separate: Illness perceptions (B-IPQ), Proactive coping (UPCC), Depressive symptoms (CES-D).

Model 2: B-IPQ, UPCC and CES-D corrected for confounders age and gender.

Model 3: B-IPQ, UPCC and CES-D additionally adjusted for dyspnea, FEV_1_, smoking status, and co-morbidities.

## Discussion

This study indicates that in COPD patients in primary care, psychological factors contribute to HRQoL. More positive perceptions about COPD and lower levels of depressive symptoms were associated with better HRQoL. Significant relations between psychological factors and DA (measured by FPI) were not found.

To appreciate the findings of this study, some limitations need to be considered. First, the cross-sectional nature of our study prohibits conclusions on causal relationships. Longitudinal data will facilitate the explanation of the relationships among psychological determinants, DA, and HRQoL in more detail. Second, the βs in the regression models were small, indicating small clinical changes per unit change.

Third, although our sample size was adequate given the research question, it did not allow us to conduct subgroup analyses per GOLD grade. Therefore, we could not describe the associations in the different stages of COPD.

The strength of the present study is its generalizability. In our study population, 60% of the patients had GOLD grade II, indicating mild to moderate COPD, which is in line with the population of COPD patients in primary care [8]. Moreover, we did not exclude patients with co-morbidities. Therefore, our study population is representative of the primary care population.

The multivariate approach (i.e., including the combination of different types of psychological factors) to explain the complexity of living with COPD represents another strength of this study.

The findings of our study are in accordance with results from previous studies concerning illness perceptions in COPD patients. Scharloo et al. [21] demonstrated that COPD patients with more positive beliefs about the effect and outcomes of their illness and with fewer strong emotional reactions to the illness had higher HRQoL scores. In a study by Fischer et al. [22], COPD patients’ beliefs about the effectiveness of medical (pharmacological) treatment in COPD were shown to be related to better outcomes in COPD.

Whereas in other patient groups, such as patients with diabetes, proactive coping has been related to better outcomes in health behavior [27,33], we could not confirm the hypothesis that COPD patients with proactive competencies have better DA and HRQoL. Possible explanations may include our relatively small sample and the mild COPD population we studied. It is unknown what this association is for COPD patients with GOLD grade III and IV, who have more potential threats to their health.

In this study, 22.2% of the patients had depressive symptoms, which is comparable to the prevalence of depressive symptoms (24.6%) in a meta-analysis study by Zhang [31]. However, contrary to other studies [39,40], daily activities were not associated with depressive symptoms in our study. This finding may partially be explained by the use of other DA and dyspnea measurement tools. Nevertheless, in line with other studies [13,41], depressive symptoms were associated with HRQoL.

The finding of our study that dyspnea contributes to HRQoL is comparable to other studies regarding the association between dyspnea and HRQoL [41-43]. Moreover, our study revealed that 60% of the variance in HRQoL was explained by the combination of illness perceptions, depressive symptoms, and dyspnea.

Although the level of daily activities of COPD patients in this study (1.80) was in line with levels of activity in other studies using the FPI in COPD patients (2.2 in a study by Kapella [44] and 1.87 in study by Wall [39]), in contrast to our hypothesis, we did not find associations between psychological factors and DA. This finding is in contrast to the results from previous studies concerning relationships between psychological factors and DA [45-47]. This finding could be explained by the use of different measurement tools for DA. Fischer et al. [20] used walking test results as the outcome parameter, in contrast to our study, which used the FPI questionnaire. A possible explanation is that a walking test measures the physical capacity or ability to participate in day-to-day activities [48,49], whereas the functional performance inventory measures self-reported activities in daily life [36].

In other studies concerning factors affecting health status [40,45], other DA measurement tools (Saint George’s Respiratory Questionnaire and the Pulmonary Functional Status Tool) were used. Obviously, other factors are likely to influence the activities people choose to perform on a daily basis, such as environmental factors, medical history, and other personal characteristics [44]. In our study, dyspnea was significantly associated with DA, which is in line with results from other studies [50,51].

## Conclusions and implications

The results of this study indicate that in COPD patients, the combination of illness perceptions and depressive symptoms contribute to HRQoL. More positive illness perceptions about COPD and lower levels of depressive symptoms were associated with better HRQoL. This study contributes to the understanding of the complexity of living with COPD.

Although small βs in the regression models indicate a small clinical contribution, these results provide starting points for the development of interventions focusing on psychological concepts to support COPD patients in their daily life and disease management. Patients’ perceptions of COPD, depressive symptoms, and dyspnea should be explored (e.g., with questionnaires), discussed with the patient, and, if necessary, corrected at an early stage. Positive (realistic) beliefs should be stimulated, and negative beliefs should be prevented or challenged [52-54]. Depressive symptoms and dyspnea should also be identified and treated properly. These approaches may result in better HRQoL in COPD patients. However, because interventions focusing on these psychological concepts have only recently been described in patients with other chronic diseases [55-57], it is important to develop and test the effectiveness of these interventions for COPD patients.

## Competing interests

The authors declare that they have no competing interests.

## Authors’ contributions

SW contributed to the study concept and design, data collection, data analysis, and writing of the manuscript and takes full responsibility for the integrity of the data and the accuracy of the data analysis. JL contributed to the study concept and design, providing input on the data analysis, reviewing, and final editing of the manuscript. RD contributed to data analysis and editing of the manuscript. MS contributed to the study concept and design, providing input on the data analysis, reviewing, and final editing of the manuscript. All authors read and approved the final manuscript.

## Authors’ information

SW is a researcher and a nurse in the Heart & Lungs Division at the University Medical Center Utrecht. As a clinical nurse specialist in lung diseases, she has extensive experience in the care and guidance of COPD patients. She is working on her PhD research project. The aim of this project is to develop and test a feasible intervention for COPD patients to be applied by practice nurses in a primary care setting. This nursing intervention is being evaluated in a cluster randomized trial (started spring 2013). In previous projects, she has gained experience with developing interventions to improve self-management and decrease functional decline.

JWL is a pulmonologist and head of the Department of Respiratory Medicine of the University Medical Center Utrecht. Among other clinical and preclinical studies on COPD, including the TiPharma projects on COPD and the COPACETIC study, a KP7 project, he has been involved in studies about patient adherence, illness perceptions, and medication adherence in patients with pulmonary diseases.

RD is a medical doctor working in the field of respiratory medicine. He has a special interest in patient adherence and illness perceptions in COPD patients.

MS is a researcher, a nurse, an appointed professor in Nursing Science at the University Medical Center Utrecht, and a professor of care for older people at the University of Applied Sciences in Utrecht. Her research focuses on the daily functioning of older people with multi-morbidity.

## References

[B1] Lozano R Naghavi M Foreman K Lim S Shibuya K Aboyans V Abraham J Adair T Aggarwal R Ahn SY AlMazroa MA Alvarado M Anderson HR Anderson LM Andrews KG Atkinson C Baddour LM Barker-Collo S Bartels DH Bell ML Benjamin EJ Bennett D Bhalla K Bikbov B Abdulhak AB Birbeck G Blyth F Bolliger I Boufous S Bucello C Global and regional mortality from 235 causes of death for 20 age groups in 1990 and 2010: a systematic analysis for the Global Burden of Disease Study 2010 Lancet 2012 380 9859 2095 2128 23245604 10.1016/S0140-6736(12)61728-0 PMC10790329

[B2] Decramer M Sibille Y Bush A Carlsen K Rabe KF Clancy L Turnbull A Nemery B Simonds A Troosters T The European Union conference on chronic respiratory disease: purpose and conclusions Eur Respir J 2011 37 4 738 742 21454890 10.1183/09031936.00020211

[B3] Pitta F Troosters T Spruit MA Probst VS Decramer M Gosselink R Characteristics of physical activities in daily life in chronic obstructive pulmonary disease Am J Respir Crit Care Med 2005 171 9 972 977 15665324 10.1164/rccm.200407-855OC

[B4] Jones PW Activity limitation and quality of life in COPD COPD 2007 4 3 273 278 17729072 10.1080/15412550701480265

[B5] Bossenbroek L De Greef MHG Wempe JB Krijnen WP ten Hacken NHT Daily physical activity in patients with chronic obstructive pulmonary disease: a systematic review COPD 2011 8 4 306 319 21728804 10.3109/15412555.2011.578601

[B6] Vorrink SN Kort HS Troosters T Lammers JW Level of daily physical activity in individuals with COPD compared with healthy controls Respir Res 2011 12 33 21426563 10.1186/1465-9921-12-33 PMC3070642

[B7] Van Den Borst B Slot IGM Hellwig VACV Vosse BAH Kelders MCJM Barreiro E Schols AMWJ Gosker HR Loss of quadriceps muscle oxidative phenotype and decreased endurance in patients with mild-to-moderate COPD J Appl Physiol 2013 114 9 1319 1328 22815389 10.1152/japplphysiol.00508.2012

[B8] Schermer T van Weel C Barten F Buffels J Chavannes N Kardas P Ostrem A Schneider A Yaman H Prevention and management of chronic obstructive pulmonary disease (COPD) in primary care: position paper of the European Forum for Primary Care Qual Prim Care 2008 16 5 363 377 18973718

[B9] Smeele I van Weel C Van Schayck CP van der Molen T Thoonen BP Schermer T Sachs APE Muris JWM Chavannes N Kolnaar BGM Grol MH Geijer RMN NHG Standaard COPD Huisarts Wet 2007 50 8 362 379

[B10] Kruis AL Chavannes NH Potential benefits of integrated COPD management in primary care Monaldi arch chest dis 2010 73 3 130 134 21214043 10.4081/monaldi.2010.297

[B11] Zakrisson AB Engfeldt P Hagglund D Odencrants S Hasselgren M Arne M Theander K Nurse-led multidisciplinary programme for patients with COPD in primary health care: a controlled trial Prim Care Respir J 2011 20 4 427 433 21687920 10.4104/pcrj.2011.00060 PMC6549878

[B12] Uijen AA Bischoff EW Schellevis FG Bor HH van den Bosch WJ Schers HJ Continuity in different care modes and its relationship to quality of life: a randomised controlled trial in patients with COPD Br J Gen Pract 2012 62 599 e422 428 22687235 10.3399/bjgp12X649115 PMC3361122

[B13] Bentsen SB Henriksen AH Wentzel-Larsen T Hanestad BR Wahl AK What determines subjective health status in patients with chronic obstructive pulmonary disease: importance of symptoms in subjective health status of COPD patients Health Qual Life Outcomes 2008 6 115 19094216 10.1186/1477-7525-6-115 PMC2640371

[B14] Arne M Lundin F Boman G Janson C Janson S Emtner M Factors associated with good self-rated health and quality of life in subjects with self-reported COPD Int J Chron Obstruct Pulmon Dis 2011 6 511 519 22069362 10.2147/COPD.S24230 PMC3206767

[B15] Disler RT Gallagher RD Davidson PM Factors influencing self-management in chronic obstructive pulmonary disease: an integrative review Int J Nurs Stud 2012 49 2 230 242 22154095 10.1016/j.ijnurstu.2011.11.005

[B16] de Ridder D Geenen R Kuijer R van Middendorp H Psychological adjustment to chronic disease Lancet 2008 372 9634 246 255 18640461 10.1016/S0140-6736(08)61078-8

[B17] Hynninen KMJ Breitve MH Wiborg AB Pallesen S Nordhus IH Psychological characteristics of patients with chronic obstructive pulmonary disease: a review J Psychosom Res 2005 59 6 429 443 16310027 10.1016/j.jpsychores.2005.04.007

[B18] McCathie HCF Spence SH Tate RL Adjustment to chronic obstructive pulmonary disease: the importance of psychological factors Eur Respir J 2002 19 1 47 53 11852894 10.1183/09031936.02.00240702

[B19] Leventhal HL Brissette I Leventhal EA Cameron LD Leventhal H Common-sense model of self-regulation of health and illness The Self-regulation of health and illness behaviour 2003 London: Routledge 42 65

[B20] Fischer MJ Scharloo M Abbink JJ Van T Hul AJ Van Ranst D Rudolphus A Weinman J Rabe KF Kaptein AA Drop-out and attendance in pulmonary rehabilitation: the role of clinical and psychosocial variables Respir Med 2009 103 10 1564 1571 19481919 10.1016/j.rmed.2008.11.020

[B21] Scharloo Margreet K Adrian S Maryanne P Harry B Elisabeth R Klaus W Emiel FM Illness perceptions and quality of life in patients with chronic obstructive pulmonary disease J asthma 2007 44 7 575 581 17885862 10.1080/02770900701537438

[B22] Fischer MJ Scharloo M Abbink J Van T Hul A Van Ranst D Rudolphus A Weinman J Rabe KF Kaptein AA Concerns about exercise are related to walk test results in pulmonary rehabilitation for patients with COPD Int J Behav Med 2012 19 39 47 21080250 10.1007/s12529-010-9130-9 PMC3277820

[B23] Scharloo M Kaptein AA Weinman J Hazes JM Willems LN Bergman W Rooijmans HG Illness perceptions, coping and functioning in patients with rheumatoid arthritis, chronic obstructive pulmonary disease and psoriasis J Psychosom Res 1998 44 5 573 585 9623878 10.1016/s0022-3999(97)00254-7

[B24] Kruis A Boland M Schoonvelde C Assendelft W Molken M Gussekloo J Tsiachristas A Chavannes N RECODE: design and baseline results of a cluster randomized trial on cost-effectiveness of integrated COPD management in primary care BMC Pulm Med 2013 13 1 17 23522095 10.1186/1471-2466-13-17 PMC3637448

[B25] GOLDfor Chronic Obstructive Lung Disease (GOLD) 2013 http://www.goldcopd.com

[B26] Aspinwall LG Taylor SE A stitch in time: self-regulation and proactive coping Psych Bull 1997 121 3 417 436 10.1037/0033-2909.121.3.417 9136643

[B27] Thoolen B de Ridder D Bensing J Gorter K Rutten G Beyond good intentions: the development and evaluation of a proactive self-management course for patients recently diagnosed with type 2 diabetes Health Educ Res 2008 23 1 53 61 17289660 10.1093/her/cyl160

[B28] Bode C de Ridder DT Kuijer RG Bensing JM Effects of an intervention promoting proactive coping competencies in middle and late adulthood Gerontologist 2007 47 1 42 51 17327539 10.1093/geront/47.1.42

[B29] Kunik ME Roundy K Veazey C Souchek J Richardson P Wray NP Stanley MA Surprisingly high prevalence of anxiety and depression in chronic breathing disorders Chest 2005 127 4 1205 1211 15821196 10.1378/chest.127.4.1205

[B30] Maurer J Rebbapragada V Borson S Goldstein R Kunik ME Yohannes AM Hanania NA ACCP Workshop Panel on Anxiety and Depression in COPD Anxiety and depression in COPD: current understanding, unanswered questions, and research needs Chest 2008 134 4 Suppl 43S 56S 18842932 10.1378/chest.08-0342 PMC2849676

[B31] Zhang MWB Ho RCM Cheung MWL Fu E Mak A Prevalence of depressive symptoms in patients with chronic obstructive pulmonary disease: a systematic review, meta-analysis and meta-regression Gen Hosp Psychiatry 2011 33 3 217 223 21601717 10.1016/j.genhosppsych.2011.03.009

[B32] Broadbent E Petrie KJ Main J Weinman J The brief illness perception questionnaire J Psychosom Res 2006 60 6 631 637 16731240 10.1016/j.jpsychores.2005.10.020

[B33] Bode C Thoolen B Ridder De D Measuring proactive coping. Psychometric characteristics of the Utrecht Proactive Coping Competence scale (UPCC) Psychol Gezondheid 2008 36 2 81 91

[B34] Ensel W Lin N, Dean A, Ensel W Measuring depression: the CES-D scale Social Support, Life Events, and Depression 1986 Orlando, Florida: Academic Press, Inc 51 70

[B35] Bestall JC Paul EA Garrod R Garnham R Jones PW Wedzicha JA Usefulness of the Medical Research Council (MRC) dyspnoea scale as a measure of disability in patients with chronic obstructive pulmonary disease Thorax 1999 54 7 581 586 10377201 10.1136/thx.54.7.581 PMC1745516

[B36] Leidy NK Psychometric properties of the functional performance inventory in patients with chronic obstructive pulmonary disease Nurs Res 1999 48 1 20 28 10029398 10.1097/00006199-199901000-00004

[B37] van der Molen T Willemse B Schokker S ten Hacken N Postma D Juniper E Development, validity and responsiveness of the clinical COPD questionnaire Health Qual Life Outcomes 2003 1 1 13 12773199 10.1186/1477-7525-1-13 PMC156640

[B38] Charlson M Szatrowski TP Peterson J Gold J Validation of a combined comorbidity index J Clin Epidemiol 1994 47 11 1245 1251 7722560 10.1016/0895-4356(94)90129-5

[B39] Wall MP Predictors of functional performance in community-dwelling people with COPD J Nurs Scholarsh 2007 39 3 222 228 17760794 10.1111/j.1547-5069.2007.00172.x

[B40] Weaver TE Richmond TS Narsavage GL An explanatory model of functional status in chronic obstructive pulmonary disease Nurs Res 1997 46 1 26 31 9024421 10.1097/00006199-199701000-00005

[B41] Moy ML Reilly JJ Ries AL Mosenifar Z Kaplan RM Lew R Garshick E National Emphysema Treatment Trial Research Group Multivariate models of determinants of health-related quality of life in severe chronic obstructive pulmonary disease J Rehabil Res Dev 2009 46 5 643 654 19882497 10.1682/JRRD.2008.09.0127 PMC2774920

[B42] Jones P Miravitlles M van der Molen T Kulich K Beyond FEV(1) in COPD: a review of patient-reported outcomes and their measurement Int J Chron Obstruct Pulmon Dis 2012 7 697 709 23093901 10.2147/COPD.S32675 PMC3476498

[B43] Tsiligianni I Kocks J Tzanakis N Siafakas N van der Molen T Factors that influence disease-specific quality of life or health status in patients with COPD: a review and meta-analysis of Pearson correlations Prim Care Respir J 2011 20 3 257 268 21472192 10.4104/pcrj.2011.00029 PMC6549844

[B44] Kapella MC Larson JL Covey MK Alex CG Functional performance in chronic obstructive pulmonary disease declines with time Med Sci Sports Exerc 2011 43 2 218 224 20543752 10.1249/MSS.0b013e3181eb6024 PMC2989985

[B45] Hynninen MJ Pallesen S Nordhus IH Factors affecting health status in COPD patients with co-morbid anxiety or depression Int J Chron Obstruct Pulmon Dis 2007 2 3 323 328 18229570 PMC2695194

[B46] Fischer M Scharloo M Abbink J Van T Hul A Van Ranst D Rudolphus A Weinman J Rabe K Kaptein AA The dynamics of illness perceptions: testing assumptions of Leventhal's common-sense model in a pulmonary rehabilitation setting Br J Health Psychol 2010 15 Pt 4 887 903 20230660 10.1348/135910710X492693

[B47] Altenburg WA Bossenbroek L De Greef MHG Kerstjens HAM Ten Hacken NHT Wempe JB Functional and psychological variables both affect daily physical activity in COPD: A structural equations model Respir Med 2013 107 11 1740 1747 23810269 10.1016/j.rmed.2013.06.002

[B48] Leidy NK Functional status and the forward progress of merry-go-rounds: toward a coherent analytical framework Nurs Res 1994 43 4 196 202 8047422

[B49] Stull DE Kline Leidy N Jones PW Ståhl E Measuring functional performance in patients with COPD: a discussion of patient-reported outcome measures Curr Med Res Opin 2007 23 11 2655 2665 17883881 10.1185/030079907x233133

[B50] Pitta F Takaki MY Oliveira NH Sant'anna TJ Fontana AD Kovelis D Camillo CA Probst VS Brunetto AF Relationship between pulmonary function and physical activity in daily life in patients with COPD Respir Med 2008 102 8 1203 1207 18573647 10.1016/j.rmed.2008.03.004

[B51] Reardon JZ Lareau SC ZuWallack R Functional status and quality of life in chronic obstructive pulmonary disease Am J Med 2006 119 10 Suppl 1 32 37 16996897 10.1016/j.amjmed.2006.08.005

[B52] Atkins CJ Kaplan RM Timms RM Reinsch S Lofback K Behavioral exercise programs in the management of chronic obstructive pulmonary disease J Consult Clin Psychol 1984 52 4 591 603 6470285 10.1037//0022-006x.52.4.591

[B53] Kaptein AA Scharloo M Fischer MJ Snoei L Cameron LD Sont JK Rabe KF Weinman J Illness perceptions and COPD: an emerging field for COPD patient management J Asthma 2008 45 8 625 629 18951252 10.1080/02770900802127048

[B54] Kaptein AA Scharloo M Fischer MJ Snoei L Hughes BM Weinman J Kaplan RM Rabe KF 50 years of psychological research on patients with COPD–road to ruin or highway to heaven? Respir Med 2009 103 1 3 11 18930645 10.1016/j.rmed.2008.08.019

[B55] Petrie KJ Cameron LD Ellis CJ Buick D Weinman J Changing illness perceptions after myocardial infarction: an early intervention randomized controlled trial Psychosom Med 2002 64 4 580 586 12140347 10.1097/00006842-200207000-00007

[B56] Davies MJ Heller S Skinner TC Campbell MJ Carey ME Cradock S Dallosso HM Daly H Doherty Y Eaton S Fox C Oliver L Rantell K Rayman G Khunti K Diabetes Education and Self Management for Ongoing and Newly Diagnosed Collaborative Effectiveness of the diabetes education and self management for ongoing and newly diagnosed (DESMOND) programme for people with newly diagnosed type 2 diabetes: cluster randomised controlled trial BMJ 2008 336 7642 491 495 18276664 10.1136/bmj.39474.922025.BE PMC2258400

[B57] Jansen DL Heijmans M Rijken M Kaptein AA The development of and first experiences with a behavioural self-regulation intervention for end-stage renal disease patients and their partners J Health Psychol 2011 16 2 274 283 20733011 10.1177/1359105310372976

